# Chikungunya virus disease outbreak in Yap State, Federated States of Micronesia

**DOI:** 10.1371/journal.pntd.0005410

**Published:** 2017-03-01

**Authors:** Daniel M. Pastula, W. Thane Hancock, Martin Bel, Holly Biggs, Maria Marfel, Robert Lanciotti, Janeen Laven, Tai-Ho Chen, J. Erin Staples, Marc Fischer, Susan L. Hills

**Affiliations:** 1 Epidemic Intelligence Service Program Office, Centers for Disease Control and Prevention (CDC), Atlanta, Georgia, United States of America; 2 Arboviral Diseases Branch, Division of Vector-Borne Diseases (DVBD), CDC, Fort Collins, Colorado, United States of America; 3 Yap State Department of Health, Colonia, Yap State, Federated States of Micronesia; 4 Rickettsial Zoonoses Branch, DVBD, CDC, Atlanta, Georgia, United States of America; 5 Division of Global Migration and Quarantine, CDC, Honolulu, Hawaii, United States of America; University of Texas Medical Branch, UNITED STATES

## Abstract

**Background:**

Chikungunya virus is a mosquito-borne alphavirus which causes an acute febrile illness associated with polyarthralgia. Beginning in August 2013, clinicians from the Yap State Department of Health in the Federated States of Micronesia (FSM) identified an unusual cluster of illness which was subsequently confirmed to be chikungunya virus disease. Chikungunya virus disease previously had not been recognized in FSM.

**Methodology/Principal findings:**

Information from patients presenting to healthcare facilities was collected and analyzed. During August 11, 2013, to August 10, 2014, a total of 1,761 clinical cases were reported for an attack rate of 155 clinical cases per 1,000 population. Among residents of Yap Main Island, 3% were hospitalized. There were no deaths. The outbreak began on Yap Main Island and rapidly spread throughout Yap Main Island and to three neighboring islands.

**Conclusions/Significance:**

Chikungunya virus can cause explosive outbreaks with substantial morbidity. Given the increasing globalization of chikungunya virus, strong surveillance systems and access to laboratory testing are essential to detect outbreaks.

## Author summary

Chikungunya virus can cause large outbreaks with substantial morbidity, especially in places with certain *Aedes* species of mosquitos and immunologically-naïve populations. In August 2013, chikungunya virus disease was identified for the first time in the Federated States of Micronesia. The explosive outbreak that followed is described in this report. Given the increasing globalization of chikungunya virus, strong surveillance systems and access to laboratory testing are essential to detect outbreaks. When outbreaks occur, a swift vector control response and implementation of prevention measures are important to limit disease spread.

## Introduction

Chikungunya virus is a mosquito-borne alphavirus capable of causing large outbreaks of acute febrile illness with severe polyarthralgia [1,2]. First identified in East Africa in 1952, as of mid-2013 chikungunya virus had caused outbreaks in Africa, Europe, Asia, and islands in the Indian and Western Pacific Oceans [1,2]. Chikungunya virus transmission previously had not been identified in the Federated States of Micronesia (FSM).

During August to October 2013, clinicians from the Yap State Department of Health in FSM identified an unusual cluster of illness characterized by acute fever and arthralgia. Among 51 patients with samples initially sent to the U.S. Centers for Disease Control and Prevention (CDC), 38 (75%) had evidence of recent chikungunya virus infection by detection of ribonucleic acid (RNA) or anti-chikungunya virus immunoglobulin (Ig) M and neutralizing antibodies. Testing for other arboviruses, including dengue, Zika, Ross River, and O’nyong-nyong viruses by reverse transcription-polymerase chain reaction (RT-PCR) or IgM tests was negative. We describe the epidemiologic features of FSM’s first known chikungunya virus disease outbreak.

## Methods

### Setting

**Fig 1:**
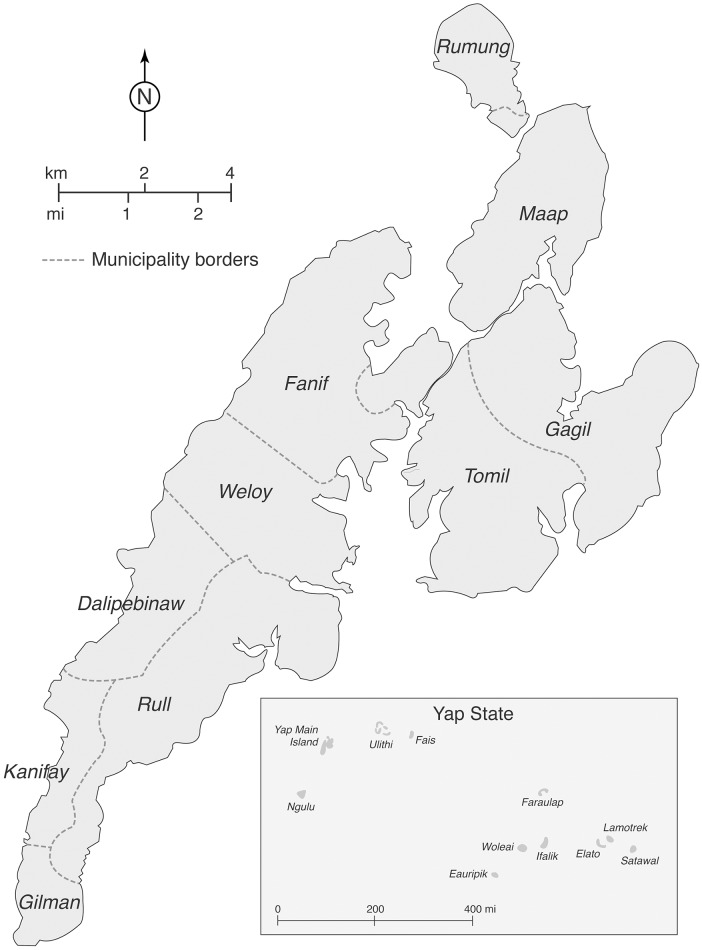
Yap Main Island and Yap State, Federated States of Micronesia.

Yap State is a collection of islands in the western part of FSM in the western Pacific Ocean. It consists of Yap Main Island, which is divided into 10 municipalities, and 10 inhabited neighboring islands and atolls (Fig 1). Yap State’s 2010 census population was 11,376 persons, with 7,370 (65%) on Yap Main Island [3]. Yap State has a public health care system with no private providers.

### Case definition

We defined a clinical case of chikungunya virus disease as a Yap State resident with acute onset of fever and new onset of arthralgia or arthritis on or after August 11, 2013 (the onset date for the first laboratory-confirmed case). A laboratory-confirmed case was a clinical case with chikungunya virus RNA or IgM antibodies detected in serum. Clinical cases who resided on an island with no laboratory-confirmed cases were excluded from the analysis. A clinical case without detectable IgM antibodies in serum collected ≥8 days after illness onset was considered not to have chikungunya virus infection.

### Case ascertainment and data collection

Cases were identified among patients presenting to any health care facility in Yap State, including Yap Memorial Hospital on Yap Main Island or any outpatient clinic located throughout Yap State. All patients presenting with fever or new onset of arthralgia or arthritis were evaluated by a healthcare provider. For patients who met the clinical case definition, health care providers on Yap Main Island completed a case report form and on neighboring islands maintained a line list with patient demographic and symptom information.

### Laboratory testing

After identification of chikungunya virus as the causative agent of the outbreak, only a subset of subsequent clinical cases had serum samples tested for chikungunya virus infection. Testing of all samples was not possible for logistical reasons. Clinical cases from new geographic areas, who were hospitalized, and those aged <5 years were prioritized. In most weeks a convenience sample of clinical cases from Yap Main Island also had samples tested.

Samples collected from patients beginning January 1, 2013, as part of routine dengue fever surveillance on Yap Main Island also were available. There samples were retrospectively tested to establish the onset date of the chikungunya virus disease outbreak as accurately as possible.

Serum samples were tested for chikungunya virus RNA byRT-PCR and/or anti-chikungunya virus IgM antibodies by enzyme-linked immunosorbent assay (ELISA). The test used depended on the timing of sample collection, with RT-PCR typically being used for samples collected ≤5 days after illness onset and IgM ELISA for samples collected ≥5 days after illness onset [4]. Nearly all chikungunya virus testing was performed at the CDC Arboviral Diseases Laboratory. One batch of samples was tested at the Institut Louis Malarde Laboratory in French Polynesia. A subset of clinical cases also had serum samples tested for dengue virus non-structural protein 1 (NS1) antigen and IgM antibodies by the Standard Diagnostics BIOLINE Dengue Duo kit.

### Statistical analyses

Data were entered into a Microsoft Access database and analyzed using Microsoft Excel 2010 and Epi Info 7.1.4. For persons who presented on two or more occasions, only data from the first visit were included. Chikungunya virus disease attack rates per 1,000 population were calculated using 2010 census data [3]. Hospitalization rates were calculated for cases on Yap Main Island where the only hospital was located.

### Ethics statement

This assessment was judged to be non-research public health practice, and therefore it was not subject to institutional review board review requirements. For epidemiological analysis, the data were anonymized.

## Results

From August 11, 2013, to August 10, 2014, a total of 1,761 clinical cases were reported to the Yap State Department of Health. This represents an attack rate of 155 clinical cases per 1,000 persons i.e., 15% of the population sought health care for a clinically-compatible illness. Overall, 904 (51%) clinical cases were female, and the median age was 30 years (range: 3 weeks–92 years). All age groups had attack rates greater than 110 per 1,000 population, with the highest attack rates in persons aged ≥30 years (Table 1). Among the 1,761 clinical cases, 1,412 (80%) resided on Yap Main Island (Table 2). The highest attack rate of 463 per 1,000 population was on the neighboring island of Fais. Among residents of Yap Main Island where the hospital was located, 3% (45/1345) were hospitalized. There were no deaths.

**Table 1:** Demographics and attack rates for clinical cases presenting to a health care facility, Yap State, August 11, 2013–August 10, 2014.

Demographics	No. cases (%) (N = 1,761)	Attack rate per 1,000 population
Sex		
Female	904 (51%)	158
Male	857 (49%)	152
Age group (years)		
0–9	264 (15%)	112
10–29	580 (33%)	140
30–49	515 (29%)	181
≥50	339 (19%)	167
Unknown	63 (4%)	—

**Table 2:** Numbers of and attack rates for clinical cases presenting to a health care facility by island, Yap State, August 11, 2013–August 10, 2014*.

Island	No. cases (%) (N = 1,761)	Attack rate per 1,000 population
Yap Main Island	1412 (80%)	192
Fais	136 (8%)	463
Ulithi^†^	122 (7%)	144
Ifalik	88 (5%)	152
Unknown	3 (<1%)	—

*Seven inhabited neighboring islands did not have any cases reported

^†^Technically an atoll

**Fig 2:**
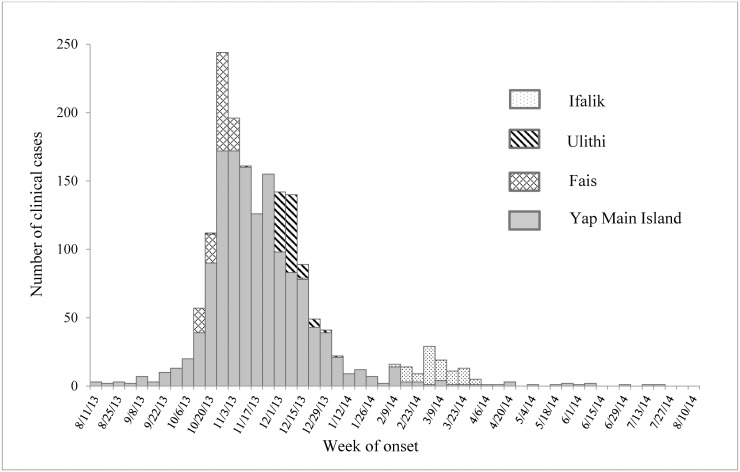
Clinical cases by week of onset, Yap State, August 11, 2013–August 10, 2014.

The outbreak began on Yap Main Island in the northeastern municipality of Tomil (Fig 1). The first reported case had no travel history, and investigations could not identify any case with an international travel history. From mid-August through the end of September 2013, 34 clinical cases had been reported from six of the 10 municipalities. The outbreak peaked in late 2013, with 1,514 (86%) of the 1,761 clinical cases occurring from October through December and clinical cases reported from all Yap Main Island municipalities and the islands of Fais and Ulithi (Fig 2). Clinical case numbers declined considerably in early 2014. However, in February 2014 cases were reported from the island of Ifalik, where transmission previously had not been documented. As of August 2014, sporadic clinical and laboratory-confirmed cases were still being reported, but after that no further laboratory testing was conducted.

Overall, 171 (10%) clinical cases across Yap State had serum samples tested and 119 (70%) had laboratory-confirmed chikungunya virus infection. Four (2%) had equivocal or indeterminate results. Forty-four (26%) had negative chikungunya virus laboratory results, but their serum samples were collected too early (<8 days) after illness onset to exclude infection. Only four (2%) clinical cases had no detectable anti-chikungunya IgM antibodies in serum collected ≥8 days after illness onset and were thus considered not to have recent chikungunya virus infection.

Two-hundred nine (12%) clinical cases had dengue testing performed by NS1 antigen or IgM antibody testing. One patient with illness onset in September had dengue virus IgM antibody detected; this patient also had chikungunya virus infection confirmed by IgM antibody testing.

Sixty-nine patients on Yap Main Island with dengue-like illness during January 1 through August 10, 2013, had samples retrospectively tested for evidence of recent chikungunya virus infection. None were positive for chikungunya virus RNA or IgM antibodies.

## Discussion

Beginning in August 2013, Yap State in FSM experienced an explosive chikungunya virus disease outbreak. Initially limited to Yap Main Island, the outbreak spread to two neighboring islands within 3 months, and to a third within 6 months. By August 2014, 15% of Yap State’s population had sought healthcare for symptoms compatible with chikungunya virus disease.

We believe this was FSM’s first chikungunya virus disease outbreak. Previous dengue and Zika virus disease outbreaks in FSM were investigated without finding evidence of chikungunya virus infections in either humans or mosquitoes [5–8]. Furthermore, all age groups had relatively high clinical disease attack rates, suggesting no prior immunity to the virus among Yap State residents. Finally, there was no laboratory evidence of chikungunya virus infection in patients with a similar clinical syndrome in the 7 months preceding this outbreak. Given these findings, it is unlikely that chikungunya virus previously was circulating in FSM.

It is unknown how chikungunya virus was introduced into Yap State, but it was likely imported by an infected person, as has occurred in previous chikungunya virus disease outbreaks [9,10]. Genetic analyses indicated the strain circulating in Yap was within the Asian genotype and closely related to strains recently isolated in China and the Philippines [11]. Travel between Yap and Southeast Asia for commerce and tourism is common, and a traveler might have been the route of introduction, with *Aedes hensilli* and *Ae*. *aegypti* mosquitoes in Yap State serving as disease vectors [12].

Previous studies have suggested considerable morbidity among patients affected by chikungunya virus disease. During an outbreak on Grand Comore Island, 52% (79/152) of ill persons missed work or school for a mean of 7 days [13]. Furthermore, some patients have persistent rheumatologic symptoms after chikungunya virus disease [1,2]. While these issues were not investigated in this outbreak, given Yap State population’s high disease attack rate, this outbreak’s impact was likely substantial.

There were two main limitations to our analysis. First, cases were limited to patients who presented to medical care and were reported to the Yap State Department of Health by a healthcare provider. Because we were unable to identify sick persons who did not present for medical care, the reported attack rate is likely an underestimate of the true rate. Second, some clinical cases might not actually have had chikungunya virus infection as not all clinical cases were tested. However, there was minimal circulation of dengue virus and no circulation of other arboviruses that cause similar clinical syndromes, and our clinical case definition was similar to one shown to have 84% sensitivity and 89% specificity during a chikungunya outbreak in Mayotte [14]. Therefore, we believe most of our clinical cases were infected with chikungunya virus.

Chikungunya virus is capable of causing explosive outbreaks with substantial morbidity, especially in places with certain *Aedes* species of mosquitos and immunologically-naïve populations. Given the increasing globalization of chikungunya virus, strong surveillance systems and access to laboratory testing are essential to detect outbreaks [15]. When outbreaks occur, swift vector control response and aggressive prevention measures (e.g., removing trash and water-collecting containers from yards, using air conditioning or ensuring window screens are intact, and applying mosquito repellant or wearing long sleeves and pants where feasible) are important to limit disease spread.
